# Prevalence and Risk Factors of Allergic Diseases Among School Students in Tabuk: A Cross-Sectional Study

**DOI:** 10.7759/cureus.36658

**Published:** 2023-03-24

**Authors:** Amirah M Alatawi, Abeer Mohammed M Alanazi, Amjad Bader S Almutairi, Raghad Faraih A Albalawi, Asmaa Abdullah M Alhakami, Aljoharh Abdulaziz S Alnuaman, Lena Defallah D Alzahrani, Ziad Saleh Albalwi, Abeer Ali H Alabawy, Lama Mueysh M Aljohani, Nouf Ali S Alatawi

**Affiliations:** 1 Department of Family and Community Medicine, Faculty of Medicine, University of Tabuk, Tabuk, SAU; 2 Department of Pediatrics, Faculty of Medicine, University of Tabuk, Tabuk, SAU

**Keywords:** saudi arabia, tabuk, risk factors, prevalence, atopic dermatitis, allergic rhinitis, bronchial asthma

## Abstract

Background: Allergic diseases such as bronchial asthma, allergic rhinitis, and atopic dermatitis are common health problems among children. The prevalence of different allergic diseases is increasing in the Kingdom of Saudi Arabia.

Objectives: This study aimed to estimate the prevalence and risk factors of allergic diseases among school students in Tabuk, Saudi Arabia.

Methods: This cross-sectional analytical study was conducted in Tabuk city, Saudi Arabia, between the first of August and the end of September, 2022. Students from primary, intermediate, and secondary schools were included. A predesigned, structured, self-administered questionnaire in the Arabic language was used to collect data.

Results: This study included 384 school students from Tabuk, Saudi Arabia. The age of the recruited students ranged from five to 19 years old. The prevalence of clinically diagnosed bronchial asthma that occurred at any time in the past was 31.8%. The prevalence of asthma symptoms was 51.0% for lifetime wheezing and 45.8% for current wheezing (in the past 12 months). The prevalence rates of clinically diagnosed allergic rhinitis and atopic dermatitis were 56.8% and 30.2%, respectively. Further, 68.2% of the school students had one or more of the diagnosed allergic diseases. The second or more childbirth orders were significantly associated with an increased risk of allergic diseases (adjusted odds ratio [AOR] = 3.140, 95% CI: 1.864-5.288). A family history of asthma or atopic conditions showed 3.118 times increased likelihood of allergic conditions (AOR = 3.118, 95% CI: 1.827-5.320). Other significant risk factors were the father’s smoking (AOR = 1.698, 95% CI: 1.024-2.817) and having a dog, cat, or bird at home (AOR = 0.493, 95% CI: 0.257-0.946).

Conclusion: The prevalence of bronchial asthma and other allergic diseases such as allergic rhinitis and atopic dermatitis among school students in Tabuk city, Saudi Arabia, is alarmingly high. Furthermore, both genetic and environmental components of allergic disease pathogenesis have been identified as risk factors.

## Introduction

Allergic diseases such as bronchial asthma, allergic rhinitis, and atopic dermatitis are highly recognized health problems among children [1]. Globally, there is an observed substantial increase in the incidence of allergic disorders due to climate changes, environmental risk factors, industrialization, and immunologic interactions [2]. Furthermore, atopy, the genetic propensity to develop immunoglobulin E (IgE) antibodies in response to exposure to an allergen, may contribute to the concomitant development of various allergies [3].

Bronchial asthma is a chronic respiratory disorder characterized by recurrent attacks of chest tightness, wheezing, coughing, and shortness of breath. Allergic rhinitis is caused by exposure of the nasal mucosa to allergens and is characterized by rhinorrhea, itching, sneezing, and sleep disorders. Atopic dermatitis is closely linked to asthma and allergic rhinitis [4].

Bronchial asthma, allergic rhinitis, and atopic dermatitis have been associated with significant morbidity, reduced quality of life, and negative consequences on school attendance, work productivity, and social and physical activities. They also constitute a high-cost burden on individuals and the healthcare system [5,6].

The prevalence of allergic diseases is on the rise in Saudi Arabia. Two million people are suffering from bronchial asthma (25% of children are affected) [7]. The prevalence of allergic rhinitis among children under the age of 15 years increased from 20% in 1986 to 25% in 1995 [8], and the overall prevalence of allergic rhinitis in Saudi Arabia is 21.2% [9].

While the exact mechanisms underlying this rapid increase in the prevalence of allergic diseases are unknown, new evidence suggests that genetic and environmental factors play an important role. Avoidance of exposure to allergens is the best treatment option. Therefore, the identification of risk factors for allergic diseases is essential [10].

The Kingdom of Saudi Arabia (KSA) comprises a variety of regions with high variability in climatic and geographical conditions. As a result, the environmental risk factors for allergic disorders, and hence the prevalence, vary on a regional scale. Therefore, this study aimed to estimate the prevalence and risk factors of allergic diseases among school students in Tabuk, Saudi Arabia.

## Materials and methods

Study design, setting, and duration

This cross-sectional analytical study was conducted in Tabuk city, Saudi Arabia, between August 1, 2022, and the end of September 2022.

Ethical considerations

The Ethical Committee of the Directorate of Health Affairs in Tabuk city, Saudi Arabia, approved the research (IRB Protocol No: TU-077/022/168). Informed consent was taken from the parents of the students before starting to fill out the questionnaire. They were informed about the objectives, risks, and benefits of the study, and they were also informed that their anonymized data will be used only for research purposes and that their confidentiality will be maintained.

Sample size and sampling technique

Raosoft (Raosoft, Inc., Seattle, Washington), an online calculator (http://www.raosoft.com/samplesize.html), was used to calculate the sample size using a margin of error of 5% and a confidence interval of 95%, assuming an average response for most of the questions of 50%. The total number of students is 211,681 according to the estimates of the General Administration of Education in Tabuk Region. The minimal required sample size is 384 participants who were recruited by random sampling technique. It was increased to 422 by 38 (10%) to compensate for a possible dropout or incomplete response.

Inclusion and exclusion criteria

Students from primary, intermediate, and secondary schools in Tabuk, Saudi Arabia, were included, while subjects who refused to participate in the study and those with incomplete data were excluded.

Questionnaire

A predesigned, structured, self-administered questionnaire in the Arabic language was adopted from Alqahtani [11] who constructed the questionnaire as a modification of the International Study of Asthma and Allergies in Childhood (ISAAC) Phase III questionnaire. They translated the questions into the Arabic terminology of Saudi Arabia, followed by back translation and assessment for face and content validity, comprehensibility, and comprehensiveness. A four-component questionnaire was used to collect the outcomes, sociodemographic, and environmental risk factors.

Procedure

We reviewed the questionnaire. Then, a pilot study was carried out on a small number of participants. Finally, the questionnaires were distributed by the students in written form to collect data from the anticipated sample of school students with the help of their parents.

Statistical analysis

All data were analyzed by the SPSS software (Statistical Package for the Social Sciences), version 26 (IBM Corp., Armonk, NY). Categorical variables were summarized as frequencies and percentages. The associations between sociodemographic and environmental variables and the development of one or more of the allergic diseases as a dependent variable were tested using X^2^ tests (Pearson's Chi-square test for independence or Fisher's exact tests as appropriate). Furthermore, all variables that showed a p-value of 0.1 or less in the univariate analysis were entered in a multivariable backward stepwise logistic regression analysis to determine the significant risk factors associated with allergic diseases. A p-value of <0.05 was considered significant.

## Results

In this study, we approached 422 school students from Tabuk, Saudi Arabia; 38 of them were excluded because of an incomplete response. The age of the recruited students ranged from five to 19 years old, with a high frequency (48.2%) of children aged between five and 11 years. Females outnumbered males (51.8% versus 48.2%, respectively). Most (96.1%) participants were Saudi. The childbirth order was either first (24.5%), second (24.0%), or third or more (51.6%). About half of the students (50.8%) belonged to primary schools, while fewer numbers were from intermediate (26.6%) or secondary (22.7%) schools. Only 11 (2.9%) of the students’ fathers and 20 (5.2%) of the mothers were illiterate. Fathers who reported smoking were 41.1%, while smoking mothers constituted 3.9%. About 155 (40.4%) participants reported a family history of asthma and other atopic conditions. Regular fast-food consumption and passing of trucks near the house were reported by 42.4% and 27.3%, respectively (Table 1).

**Table 1:** Baseline characteristics of the study participants (N = 384) N: Number; SR: Saudi Riyal.

		N = 384	%
Child age (years)	5-11	185	48.2%
	12-15	114	29.7%
	16-19	85	22.1%
Child sex	Female	199	51.8%
	Male	185	48.2%
Childbirth order	First	94	24.5%
	Second	92	24.0%
	Third or greater	198	51.6%
School type	Primary	195	50.8%
	Intermediate	102	26.6%
	Secondary	87	22.7%
Nationality	Saudi	369	96.1%
	Non-Saudi	15	3.9%
Monthly family income (SR)	<5,000	44	11.5%
	5,000-10,000	151	39.3%
	>10,000	189	49.2%
Level of father education	Illiterate	11	2.9%
	Preuniversity level	170	44.3%
	University degree	141	36.7%
	Postgraduate degree	62	16.1%
Level of mother education	Illiterate	20	5.2%
	Preuniversity level	95	24.7%
	University degree	202	52.6%
	Postgraduate degree	67	17.4%
Number of children in the family	One	26	6.8%
	Two	67	17.4%
	Three or more	291	75.8%
Type of housing	Private housing (1–3 rooms)	49	12.8%
	Private housing (4–5 rooms)	155	40.4%
	Private housing (6–7 rooms)	180	46.9%
Father’s occupation related to the health sector	No	320	83.3%
	Yes	64	16.7%
Mother’s occupation related to the health sector	No	329	85.7%
	Yes	55	14.3%
Father smoking	No	226	58.9%
	Yes	158	41.1%
Mother smoking	No	369	96.1%
	Yes	15	3.9%
Number of smokers in the household	No smokers	203	52.9%
	One or more smokers	181	47.1%
Family history of asthma and other atopic conditions	No	229	59.6%
	Yes	155	40.4%
Having a dog, cat, or bird at home	No	290	75.5%
	Yes	94	24.5%
Fast-food consumption	Regular	163	42.4%
	Rare	221	57.6%
Trucks passing near the house	Regular	105	27.3%
	Rare	279	72.7%

The present study shows that the prevalence of clinically diagnosed bronchial asthma that occurred at any time in the past was 31.8%. The prevalence of asthma symptoms was 51.0% for lifetime wheezing, 45.8% for current wheezing (in the past 12 months), and 34.9% and 52.6% for exercise-induced wheezing and nocturnal cough, respectively, over the last 12 months. Furthermore, the prevalence rates of clinically diagnosed allergic rhinitis (56.8%), lifetime sneezing (73.4%), and sneezing that occurred over the last 12 months (64.1%) were calculated. Concerning atopic dermatitis, the participants reported a 30.2% frequency of clinically diagnosed atopic dermatitis, 31.5% of recurrent rash at any time in the past, and 27.9% of recurrent rash over the past 12 months. The prevalence of an itchy rash that was coming and going for at least six months was 30.7% and that of an itchy rash over the past 12 months was 31.0% (Table 2).

**Table 2:** Prevalence of symptoms of the diagnosed and current allergic diseases among the study participants (N = 384) ^a^Ever means occurrence at any time in the past. N: Number; CI: Confidence interval.

	N = 384	%	95% CI	
			Lower	Upper
Ever asthma^a^	122	31.8%	27.3%	36.6%
Last 12 months of wheeze	176	45.8%	40.9%	50.8%
Ever wheeze^a^	196	51.0%	46.0%	56.0%
Last 12 months of exercise-induced wheeze	134	34.9%	30.3%	39.8%
Last 12 months of nocturnal cough not related to cold or chest infection	202	52.6%	47.6%	57.6%
Ever allergic rhinitis^a^	218	56.8%	51.8%	61.7%
Last 12 months sneezing	246	64.1%	59.2%	68.7%
Ever sneezing^a^	282	73.4%	68.9%	77.7%
Last 12 months of nose problems accompanied by itchy, watery eyes	206	53.6%	48.6%	58.6%
Ever atopic dermatitis^a^	116	30.2%	25.8%	34.9%
Ever recurrent rash^a^	121	31.5%	27.0%	36.3%
Last 12 months of recurrent rash	107	27.9%	23.6%	32.5%
Itchy rash that was coming and going for at least six months	118	30.7%	26.3%	35.5%
Last 12 months of itchy rash	119	31.0%	26.5%	35.7%

**Figure 1:**
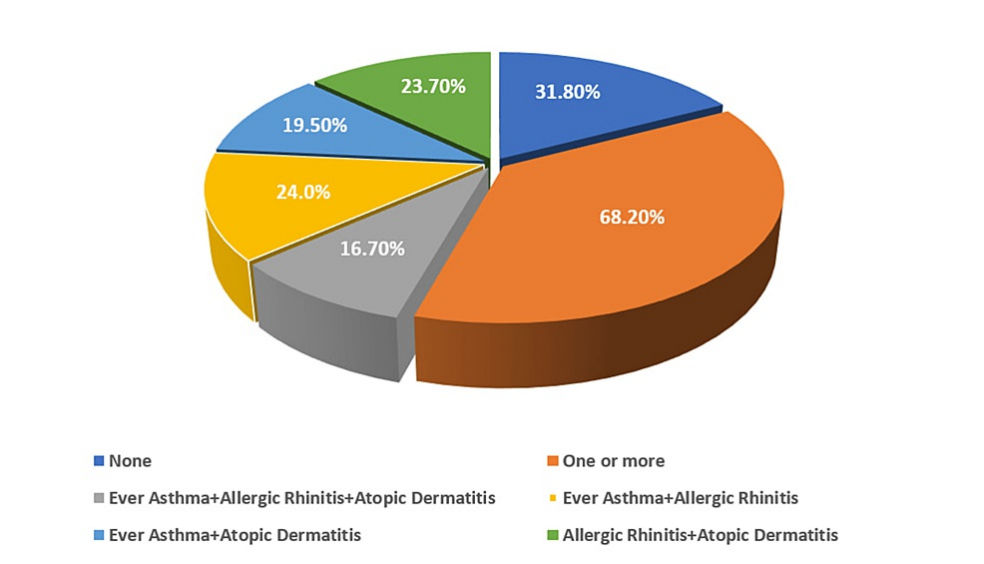
The interrelated prevalence of the diagnosed allergic diseases

Figure 1 shows that 68.2% of the school students had one or more of the diagnosed allergic diseases including bronchial asthma, allergic rhinitis, and atopic dermatitis. A few participants (16.7%) reported having the three allergic conditions, while 31.8% had no allergic diseases. The prevalence of bronchial asthma concomitant with allergic rhinitis was 24%, while the prevalence of bronchial asthma concomitant with atopic dermatitis was 19.5%. On the other hand, the interrelated occurrence of allergic rhinitis with atopic dermatitis was 23.7%.

The questions inquiring about the severity of the allergic diseases revealed that most students (54.9%) had no attacks of wheezing, and 121 (31.5%) had one to three attacks over the past 12 months. Severe bronchial asthma with more than 12 attacks of wheezing was only reported by three (0.8%) students. Wheezing that disturbed the child's sleep was either less than one night per week (28.6%) or one or more nights per week (13.8%). Regarding the symptoms of atopic dermatitis, the itchy rash affected the folds of the elbows; behind the knees; front of the ankles; under the buttocks; and around the neck, ears, or eyes in 111 (28.9%) participants. Moreover, the itchy rash kept the child awake at night for less than one night per week (19.0%) or one or more nights per week (5.5%). Nevertheless, most participants (69.5%) described that the rash cleared completely at any time during the last 12 months (Table 3).

**Table 3:** Severity of allergic diseases among the study participants (N = 384) N: Number.

		N = 384	%
How many attacks of wheezing has your child had in the last 12 months?	1 to 3	121	31.5%
	4 to 12	49	12.8%
	More than 12	3	0.8%
	None	211	54.9%
In the last 12 months, how often, on average, has your child's sleep been disturbed due to wheezing?	Less than one night per week	110	28.6%
	One or more nights per week	53	13.8%
	Never woken with wheezing	221	57.6%
Has this itchy rash at any time affected any of the following places: the folds of the elbows, behind the knees, in front of the ankles, under the buttocks, or around the neck, ears, or eyes?	No	273	71.1%
	Yes	111	28.9%
At what age did this itchy rash first occur?	Under 2 years	219	57.0%
	Ages 2 to 4	74	19.3%
	Ages 5 or more	91	23.7%
Has this rash cleared completely at any time during the last 12 months?	No	117	30.5%
	Yes	267	69.5%
In the last 12 months, how often, on average, have the child been kept awake at night by this itchy rash?	Less than one night per week	73	19.0%
	One or more nights per week	21	5.5%
	Never in the last 12 months	290	75.5%

Table 4 demonstrates that the development of one or more of the diagnosed allergic diseases including bronchial asthma, allergic rhinitis, and atopic dermatitis was significantly associated with some sociodemographic and environmental factors such as the child’s age; childbirth order; the number of children in the family; the parent's education level; parents working in the health sector; family history of asthma and other atopic conditions; school type; type of housing; having a dog, cat, or bird at home; fast-food consumption; and regular passing of trucks near the house (all p-values < 0.05). On the other hand, the child’s sex did not show a significant association with allergic diseases (p = 0.088).

**Table 4:** Sociodemographic and environmental factors associated with allergic diseases *Significant at p < 0.05. N: Number; SR: Saudi Riyal.

		Allergic diseases				Chi-square or Fisher’s exact test
		None, N = 122 (31.8%)		One or more, N = 262 (68.2%)		
		N	%	N	%	P-value
Child’s age (years)	5-11	70	57.4%	115	43.9%	0.041*
	12-15	28	23.0%	86	32.8%	
	16-19	24	19.7%	61	23.3%	
Child sex	Female	71	58.2%	128	48.9%	0.088
	Male	51	41.8%	134	51.1%	
Childbirth order	First	48	39.3%	46	17.6%	<0.001*
	Second or third	74	60.7%	216	82.4%	
School type	Primary	73	59.8%	122	46.6%	0.040*
	Intermediate	24	19.7%	78	29.8%	
	Secondary	25	20.5%	62	23.7%	
Nationality	Saudi	116	95.1%	253	96.6%	0.458
	Non-Saudi	6	4.9%	9	3.4%	
Monthly family income (SR)	Less than 5,000	19	15.6%	25	9.5%	0.183
	5,000-10,000	43	35.2%	108	41.2%	
	More than 10,000	60	49.2%	129	49.2%	
Level of father education	Illiterate	5	4.1%	6	2.3%	0.004*
	Preuniversity level	67	54.9%	103	39.3%	
	University degree	40	32.8%	101	38.5%	
	Postgraduate degree	10	8.2%	52	19.8%	
Level of mother education	Illiterate	7	5.7%	13	5.0%	<0.001*
	Preuniversity level	35	28.7%	60	22.9%	
	University degree	74	60.7%	128	48.9%	
	Postgraduate degree	6	4.9%	61	23.3%	
Number of children in the family	One	14	11.5%	12	4.6%	0.041*
	Two	19	15.6%	48	18.3%	
	Three or more	89	73.0%	202	77.1%	
Type of housing	Private housing (1–3 rooms)	25	20.5%	24	9.2%	0.005*
	Private housing (4–5 rooms)	49	40.2%	106	40.5%	
	Private housing (6–7 rooms)	48	39.3%	132	50.4%	
Father’s occupation related to the health sector	No	115	94.3%	205	78.2%	<0.001*
	Yes	7	5.7%	57	21.8%	
Mother’s occupation related to the health sector	No	111	91.0%	218	83.2%	0.043*
	Yes	11	9.0%	44	16.8%	
Father smoking	No	80	65.6%	146	55.7%	0.068
	Yes	42	34.4%	116	44.3%	
Mother smoking	No	120	98.4%	249	95.0%	0.159
	Yes	2	1.6%	13	5.0%	
Number of smokers in the family	No smokers	65	53.3%	138	52.7%	0.912
	One or more smokers	57	46.7%	124	47.3%	
Family history of asthma and other atopic conditions	No	96	78.7%	133	50.8%	<0.001*
	Yes	26	21.3%	129	49.2%	
Having a dog, cat, or bird at home	No	106	86.9%	184	70.2%	<0.001*
	Yes	16	13.1%	78	29.8%	
Fast-food consumption	Regular	39	32.0%	124	47.3%	0.005*
	Rare	83	68.0%	138	52.7%	
Trucks passing near the house	Regular	18	14.8%	87	33.2%	<0.001*
	Rare	104	85.2%	175	66.8%	

Multivariable logistic regression analysis revealed a model of risk factors that significantly contributed to the development of one or more of the diagnosed allergic diseases including bronchial asthma, allergic rhinitis, and atopic dermatitis, with an accuracy of 72.1%. The second or more childbirth orders were significantly associated with an increased risk of allergic diseases (adjusted odds ratio [AOR] = 3.140, 95% CI: 1.864-5.288). A family history of asthma or atopic conditions showed 3.118 times increased likelihood of allergic conditions (AOR = 3.118, 95% CI: 1.827-5.320). Other significant risk factors were the father’s smoking (AOR = 1.698, 95% CI: 1.024-2.817) and the presence of a dog, cat, or bird at home (AOR = 0.493, 95% CI: 0.257-0.946) (Table 5).

**Table 5:** Multivariable backward stepwise logistic regression analysis for determining the risk factors associated with allergic diseases *Significant at p < 0.05. AOR: Adjusted odds ratio; CI: Confidence interval.

Risk factors	Beta coefficient	P-value	AOR	95% CI for AOR		Accuracy	P-value
				Lower	Upper		
Childbirth order (second or greater)	1.144	<0.001*	3.140	1.864	5.288	72.1%	<0.001*
Family history of asthma or atopy	1.137	<0.001*	3.118	1.827	5.320		
Father’s smoking	0.530	0.040*	1.698	1.024	2.817		
Having a dog, cat, or bird at home	0.707	0.034*	0.493	0.257	0.946		
Constant	2.861	<0.001*	17.479				

## Discussion

This study evaluated the self-reported prevalence of one or more allergic diseases among school students in Tabuk, Saudi Arabia. Additionally, this study investigated the potential risk factors of these allergies.

In this study, the prevalence of clinically diagnosed bronchial asthma was 31.8% and that of bronchial asthma concomitant with allergic rhinitis was 24%, while the rate of bronchial asthma concomitant with atopic dermatitis was 19.5%. The study also revealed a high and alarming prevalence of wheeze ever (51.0%) and current wheeze over the past 12 months (45.8%). The prevalence of clinically diagnosed allergic rhinitis was considerably higher (56.8%), and the interrelated occurrence of allergic rhinitis with atopic dermatitis was 23.7%, while clinically diagnosed atopic dermatitis was recorded by 30.2%. There were much higher proportions (68.2%) of the school students who had one or more of these allergies. The current data showed the continuing rise of clinically diagnosed asthma and other allergic diseases among schools in Saudi Arabia.

In this comprehensive study, we estimated the prevalence of allergic disease in all age groups (5-19 years). Alternatively, previous epidemiologic studies inspecting allergic disorders among Saudi school children have focused on children of certain age groups. For example, Nahhas et al. [12] assessed the same among students aged 6-12 years in Madinah city, Saudi Arabia, while Al Ghobian et al. [13] included adolescents aged 16-18 years. An epidemiological study in KSA reported a noticeable increase in the prevalence of asthma, up to 23%, among school children aged 8-16 years [8]. A study in the Abha region reported lower figures for asthma prevalence (9%) [14], and a nationwide study in the KSA reported 8.2% self-reported asthma prevalence among adolescents [15]. In Riyadh city, the prevalence of asthma ranged from 4.5% to 19.6% in secondary school students aged 16-18 years [16]. In the Taif area, asthma prevalence among school-age children was 14.4% [17]. A corresponding study that the prevalence of asthma was 27.5% among students aged 7-19 years [11]. Recently, Alshammrie et al. [18] concluded a 12.0% prevalence of eczema among preschool-aged children in Hail City, Saudi Arabia.

In this study, the detected high prevalence of asthma symptoms like wheezing ever (51.0%) and current wheezing over the past 12 months (45.8%) without a certain diagnosis of bronchial asthma is distressing, and it reflects poor awareness of asthma diagnosis, which would be associated with inadequate asthma care. In contrast, a previous ISAAC-phase III global study that included 98 countries reported a lower mean prevalence of current wheeze among children aged six to seven years (11.7%) [19].

In the current study, the prevalence of clinically diagnosed allergic rhinitis was considerably high (56.8%). This finding was much higher than the detected prevalence of allergic rhinitis (27.1%) among Saudi school children in Jazan Region, Saudi Arabia [20]. A lower prevalence of allergic rhinitis (10%) was also reported in a multicenter study that involved Egypt, Lebanon, Saudi Arabia, Iran, and the United Arab Emirates [21].

The clinically diagnosed atopic dermatitis in this study was 30.2%. This is comparable to the reported prevalence among children aged six to seven years and 13-14 years in the United Arab Emirates [4], but it is higher than the previously reported prevalence of diagnosed atopic dermatitis in Kuwait (12%) [22].

This survey explored a high prevalence (68.2%) of one or more of the studied allergies among school children, a 24% prevalence of bronchial asthma concomitant with allergic rhinitis, a 19.5% prevalence of bronchial asthma concomitant with atopic dermatitis, and an interrelated occurrence of allergic rhinitis with atopic dermatitis of 23.7%. Further, a few participants (16.7%) reported having the three allergic conditions. A previous meta-analysis documented the overlap between bronchial asthma and other allergic diseases, with only a minority of children suffering from all three atopic disorders [23]. The co-occurrence of these allergies carries a greater risk of negative socioeconomic consequences and a lower quality of life as compared with children who have only one disorder [11]. Therefore, this distinct group of children needs further consideration from clinicians and researchers for better control and prognosis.

The severity and frequency of asthma symptoms are inconstant and are related to the extent of airflow obstruction and underlying inflammation in addition to the parent’s awareness and adherence to treatment [7]. Most students in this study reported having no attacks of wheezing, while 31.5% reported one to three attacks over the past 12 months. The students also conveyed sleep disturbance with a frequency of either less than one night per week (28.6%) or one or more nights per week (13.8%).

The observed regional differences in allergic disease estimates impose the need to investigate the specific risk factors to apply tailored intervention strategies [24]. The present survey revealed that fathers’ smoking and having a cat or dog at home are major environmental risk factors associated with a greater risk for the development of one or more of the allergic diseases. Other risk factors that increase susceptibility to asthma and other allergies were a family history of asthma or other atopic conditions and the child being the second or more in birth order.

Previous studies revealed parts of the detected risk factors in our study. Alqahtani [11] reported having a dog or cat at home as a potential risk factor for allergic diseases. Almatroudi et al. [25] investigated the factors associated with the high prevalence of respiratory allergies among adults in the KSA and found that a family history of the disease plays an important role in asthma and allergic rhinitis development. Subjects who reported a family history of bronchial asthma and allergic rhinitis were over four times and eight times more likely to have the disease compared to the individuals without a such family history. A meta-analysis of environmental and host-related risk factors associated with asthma in the Asian population concluded that a family history of asthma was the most frequently reported risk factor suggesting a great contribution of genetic components in asthma pathogenesis [26]. However, environmental factors outweigh genetic factors in determining whether an atopic individual will develop an allergic disease [27]. Mak et al. [28] stated that asthmatic patients who have parents or close friends who smoke are more likely to have asthma-associated symptoms. Likewise, Thacher et al. [29] concluded that exposure to tobacco smoke in infancy was associated with an increased risk of sensitization to food allergens and the development of eczema.

Limitations

This questionnaire base is more prone to subjectivity. However, the ISAAC core questionnaire has been broadly validated for the diagnosis and surveillance of allergic diseases.

## Conclusions

The prevalence of bronchial asthma and other allergic diseases such as allergic rhinitis and atopic dermatitis among school students in Tabuk city, Saudi Arabia, is alarmingly high. Likewise, there is a high prevalence of lifetime wheezing with no definite diagnosis of asthma. The prevalence of bronchial asthma concomitant with allergic rhinitis or atopic dermatitis and the interrelated occurrence of allergic rhinitis with atopic dermatitis are also high. Furthermore, both genetic and environmental components of allergic disease pathogenesis have been identified as risk factors. The family history of asthma or other atopic conditions, the second or more birth order of the child, the father’s smoking, and having pets at home were associated with an increased likelihood for the development of one or more of the allergic diseases. In light of these findings, there is a need for tailored intervention programs and funding asthma clinics at school for early detection and timely management to reduce the burden of allergic diseases. Increasing awareness about childhood allergic diseases is highly needed.
